# Two New Piperazine-Triones from a Marine-Derived *Streptomycetes* sp. Strain SMS636

**DOI:** 10.3390/md17030186

**Published:** 2019-03-21

**Authors:** Xiuli Xu, Jiahui Han, Rui Lin, Steven W. Polyak, Fuhang Song

**Affiliations:** 1 School of Ocean Sciences, China University of Geosciences, Beijing 100083, China; xuxl@cugb.edu.cn (X.X.); 15632779760@163.com (J.H.); linrui520@126.com (R.L.); 2 CAS Key Laboratory of Pathogenic Microbiology and Immunology, Institute of Microbiology, Chinese Academy of Sciences, Beijing 100101, China; 3 School of Pharmacy and Medical Sciences, University of South Australia, Adelaide 5000, Australia; steven.polyak@unisa.edu.au

**Keywords:** marine-derived *Streptomycetes*, piperazine-trione, antibacterial, MRSA

## Abstract

Two new piperazine-triones lansai E and F (**1**, **2**), together with four known secondary metabolites lansai D (**3**), 1-*N*-methyl-(*E,Z*)-albonoursin (**4**), imidazo[4,5-*e*]-1,2,4-triazine (**5**), and streptonigrin (**6**) were isolated from a deep-sea-derived *Streptomycetes* sp. strain SMS636. The structures of the isolated compounds were confirmed by comprehensive spectroscopic analysis, including HRESIMS, 1D and 2D NMR. Compound **4** exhibited moderate antibacterial activities against *Staphylococcus aureus* and methicillin resistant *S. aureus* (MRSA) with Minimum Inhibitory Concentration (MIC) values of 12.5 and 25 μg/mL, respectively. Compound **6** displayed significant antibacterial activities against *S. aureus*, MRSA and Bacillus Calmette-Guérin (BCG) with MIC values of 0.78, 0.78 and 1.25 μg/mL, respectively.

## 1. Introduction

Natural products have proved to be a valuable source of new chemical entries in many therapeutic areas [1]. Piperazine-triones are a rare class of natural products produced by fungi and actinomycetes. To date, only eleven polyketides bearing piperazine-triones motif have been discovered in nature, including dithiodioxopiperazine [2,3], cytotoxic 12-demethyl-12-oxodehydroechinulin [4] and gliocladine C [5], lasiodiplines A [6], MPC 1001F and MPC 1001H [7], antiviral rubrumline L [8], neoechinuline [9], variecolorin J [10], *S*-1, 4-Dimethyl-6-[4-(3-methyl-but-2-enyloxy)-benzyl]-6-methylsulfanylpiperazine-2,3,5-trione and *R*-6-[4-(3-methyl-but-2-enyloxy)-benzyl]-6-methylsulfanyl-piperazine-2,3,5-trione [11].

**Figure 1:**
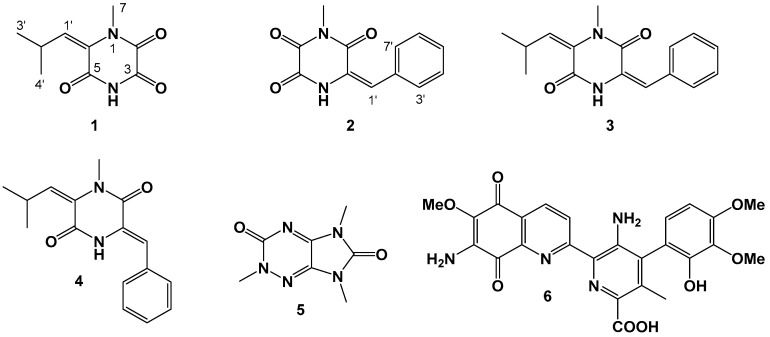
Chemical structures of **1**–**6.**

Actinomycetes characterized from the marine environment have been reported to be an excellent source for their potential to produce secondary metabolites with novel structures [12,13,14]. During the course of our ongoing efforts to discover antimicrobial secondary metabolites from marine-derived microorganisms, a crude extract from a *Streptomycetes* sp. strain SMS636 (isolated from a sediment sample collected at a depth of −3000 m from the South China Sea) exhibited significant antibacterial activity against *S. aureus*. Further chemical investigation on the fermentation material resulted in the identification of two new piperazine-triones, named as lansai E and F (**1** and **2**), together with four previously reported metabolites, lansai D (**3**) [15], 1-*N*-methyl-(*E,Z*)-albonoursin (**4**) [16], imidazo[4,5-*e*]-1,2,4-triazine (**5**) [17] and streptonigrin (**6**) [18]. Lansai E and F belong to a rather rare class of alkaloids which bears the piperazine-trione motif. The structures (Figure 1) of the isolated compounds were characterized based on comprehensive spectroscopic data, and the geometric configurations of compounds **1**–**4** were assigned by Rotating Frame Overhauser Effect Spectroscopy (ROESY) analysis. All these compounds were tested for their antimicrobial activities against *S. aureus*, methicillin resistant *S. aureus* (MRSA), *Escherichia coli*, *Pseudomonas aeruginosa*, Bacillus Calmette-Guérin (BCG), and *Candida albicans*. Compound **4** exhibited moderate antibacterial activities against *S. aureus* and MRSA, and compound **6** showed significant antibacterial activities against *S. aureus*, MRSA and BCG.

## 2. Results

### 2.1. Structure Elucidation

**Figure 2:**
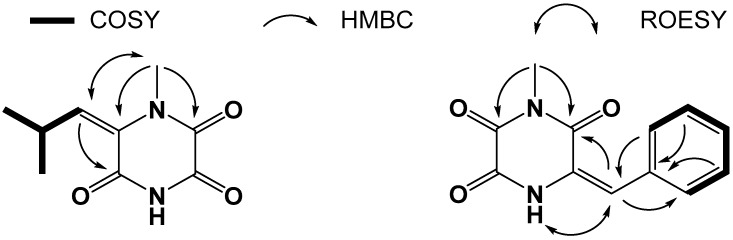
Key 2D NMR correlations for **1** and **2.**

Compound **1** was obtained as a colorless amorphous powder, and its molecular formula was established as C_9_H_12_N_2_O_3_ from the HRESIMS at *m*/*z* 219.0748 [M + Na]^+^ (calculated for C_9_H_12_N_2_O_3_Na, 219.0740, Δmmu + 0.8), accounting for five degrees of unsaturation. The ^1^H NMR spectrum (Supplementary Materials Figure S1) of **1** (Table 1) showed signals for one isopropyl at *δ*_H_ 3.94 (1H, m, H-2′) and 1.05 (6H, d, *J* = 6.6 Hz, H_3_-3′ and H_3_-4′), one singlet methyl at *δ*_H_ 3.17 (3H, s, H_3_-7), one olefinic proton at *δ*_H_ 5.81 (1H, d, *J* = 9.0 Hz, H-1′), as well as one proton at *δ*_H_ 12.01 (1H, brs) attached to *N*-4. The ^13^C NMR and HSQC data (Supplementary Materials Figures S2 and S3) for **1** revealed the carbon signals associated with the above structural units, one olefinic carbon at *δ*_C_ 127.8 (C-6), as well as three carbonyl carbons at *δ*_C_ 152.4 (C-2), 155.6 (C-3) and 160.2 (C-5). The COSY correlations (Figure 2 and Supplementary Materials Figure S4) of **1** revealed the connection from 1′ to 2′, and from 2′ to both of 3′ and 4′. The HMBC correlations (Supplementary Materials Figure S5) from H_3_-7 to C-2 and C-6 revealed the connection from C-7 to *N*-1. Crossing peaks from H-1′ to C-5 and C-6 confirmed the connection between C-5 and C-6. Using the molecular formula data and spectroscopic analysis, the structure of **1** was assigned as shown in Figure 1. The geometric configuration of the double bond was assigned as the *E* configuration by the ROESY correlation (Supplementary Materials Figure S6) from H_3_-7 to H-1′.

Compound **2** was obtained as a colorless amorphous powder, and its molecular formula was established as C_12_H_10_N_2_O_3_ from the HRESIMS at *m*/*z* 483.1274 [2M + Na]^+^ (calculated for C_24_H_20_N_4_O_6_Na, 483.1275, Δmmu − 0.1), accounting for nine degrees of unsaturation. The ^1^H NMR spectrum (Supplementary Materials Figure S7) of **1** showed signals for one monosubstituted benzene at *δ*_H_ 7.53 (2H, d, *J* = 7.8 Hz, H-3′ and H-7′), 7.34 (2H, dd, *J* = 7.8, 7.8 Hz, H-4′ and H-6′) and 7.31 (1H, dd, *J* = 7.8, 7.8 Hz, H-5′), one singlet methyl at *δ*_H_ 3.07 (3H, s, H_3_-7), one olefinic proton at *δ*_H_ 6.77 (1H, s, H-1′), as well as one proton (*δ*_H_ 11.44, 1H, s) attached to *N*-4. The ^13^C NMR and HSQC data (Supplementary Materials Figures S8 and S9) for **2** revealed the carbon signals associated with the above structural units (Table 1), one olefinic carbon at *δ*_C_ 125.9 (C-3), as well as three carbonyl signals at *δ*_C_ 158.7 (C-2), 151.6 (C-5), and 156.7 (C-6). The COSY correlations (Supplementary Materials Figure S10) of **2** revealed the monosubstituted benzene ring. The HMBC correlations (Supplementary Materials Figure S11) from H_3_-7 to C-2 and C-6 revealed the connection from C-7 to *N*-1. Crossing peaks from H-1′ to C-2, C-3, C-3′ and C-7′ indicated a connection from C-3 to C-2′ through C-1′. Subsequently, the structure of **2** was assigned as shown in Figure 1. As observed for **1**, the geometric configuration of the double bond was assigned as the *E* configuration by the ROESY correlation (Supplementary Materials Figure S12) from H-4-*N* to H-1′.

### 2.2. Biological Activity

Compounds **1**–**6** were evaluated against *S. aureus* (ATCC 6538), MRSA (ATCC 29213), *E. coli* (ATCC 11775), *P. aeruginosa* (ATCC 15692), BCG, and *C. albicans* (ATCC 10231). Compound **4** showed moderate antibacterial activities against *S. aureus* and MRSA with Minimum Inhibitory Concentration (MIC) values of 12.5 and 25 μg/mL, respectively. Compound **6** also exhibited significant antibacterial activities against *S. aureus*, MRSA, and BCG with MIC values of 0.78, 0.78 and 1.25 μg/mL (Table 2). None of the tested compounds displayed significant antimicrobial activities against *E. coli*, *P. aeruginosa*, and *C. albicans* at 100 µg/mL, suggesting that the spectrum of activities for the active compounds is likely to be restricted to Gram-positive bacteria.

## 3. Materials and Methods

### 3.1. General Experimental Procedures

NMR spectra were obtained on a Bruker Avance DRX600 spectrometer (Bruker BioSpin AG, Fällanden, Switzerland) with residual solvent peaks as references (DMSO-*d*_6_: δ_H_ 2.50, δ_C_ 39.52). High-resolution ESIMS measurements were obtained on a Bruker micrOTOF mass spectrometer (Bruker Daltonics, Billerica, MA, USA) by direct infusion in MeCN at 3 mL/min using sodium formate clusters as an internal calibrate. HPLC was performed using an Agilent 1100 Series separations (Agilent Technology, Inc., Waldbronn, Germany) module equipped with Agilent 1100 Series diode array, Agilent 1100 Series fraction collector, and Agilent SB-C18 column (250 × 9.4 mm, 5 µm).

### 3.2. Microbial Material

The *Streptomycetes* sp. strain SMS636 used as the producing strain was isolated from a sediment sample collected from the South China Sea and grown on an ISP2 agar slant consisting of glucose 0.4%, yeast extract 0.4%, malt extract 1.0%, agar 2.0% (pH 7.2). This strain was identified as *Streptomycetes* sp. based on phylogenetic analysis of 16S rRNA gene sequence (Supplementary Materials Figure S13). The 16S rRNA sequence of SMS636 was assigned to the GenBank accession number MK334651.

### 3.3. Fermentation and Extraction

A stock culture of the producing strain was grown and maintained on ISP2 agar slant. The stock culture was transferred into 250-mL Erlenmeyer flasks containing 40 mL of seed medium (ISP2 liquid medium), and the flasks were incubated on a rotary shaker (200 rpm) at 28 °C for 96 h. 10 mL of the seed culture was inoculated into 1,000 mL Erlenmeyer flasks containing 250 mL of the producing medium (glucose 0.5%, lactose 4%, cotton seed protein 3%, Bacto Peptone 0.5%, K_2_HPO_4_ 0.05%, MgSO_4_ 7H_2_O 0.05%, and KCl 0.03%, pH 7.0), and the flasks were incubated at 28 °C with shaking (140 rpm) for 10 days. The culture broths were combined and centrifuged to yield supernatant and mycelial fractions. The supernatant was partitioned with equal volume of EtOAc (×3) and the solvent was evaporated under reduced pressure to obtain crude extract F1 (520 mg). The mycelial was extracted by 500 mL acetone (×3) and the solvent was evaporated under reduced pressure to afford crude extract F2 (340 mg).

### 3.4. Isolation and Purification

The crude extract F1 and F2 were then sequentially triturated with hexane (3 × 10 mL), DCM (3 × 10 mL) and MeOH (3 × 10 mL), respectively, then concentrated in vacuo, to afford F1-1 (175 mg), F1-2 (102 mg), F1-3 (65 mg), F2-1 (88 mg), F2-2 (58 mg), and F2-3 (42 mg), respectively. F1-2 was subjected to HPLC fractionation (Agilent SB-C18, 250 × 9.4 mm column, 5 μm, 3.0 mL/min, gradient elution from 20–100% MeCN/H_2_O over 15 min with a hold at 100% MeCN for 5 min and with isocratic 0.01% TFA modifier) to yield compounds **1** (t_R_ = 7.8 min, 0.8 mg), **2** (t_R_ = 9.1 min, 1.1 mg) and **3** (t_R_ = 13.0 min, 1.3 mg). F2-1 was subjected to HPLC fractionation (Agilent SB-C18, 250 × 9.4 mm column, 5 μm, 3.0 mL/min, gradient elution from 20–100% MeCN/H_2_O over 15 min with a hold at 100% MeCN for 5 min and with isocratic 0.01% TFA modifier) to yield compounds **4** (t_R_ = 13.9 min, 2.3 mg), **5** (t_R_ = 5.0 min, 1.4 mg) and **6** (t_R_ = 12.1 min, 3.2 mg).

#### 3.4.1. Lansai E (1)

Colorless amorphous powder; UV (MeOH) λ_max_ (logε) 234 (3.60), 304 (3.34) nm; (+)-ESIMS *m*/*z* 197.1 [M + H]^+^; (+)-HRESIMS *m*/*z* 219.0748 [M + Na]^+^ (calcd. For C_9_H_12_N_2_O_3_Na, 219.0740).

#### 3.4.2. Lansai F (2)

Colorless amorphous powder; UV (MeOH) λ_max_ (logε) 341 (3.57) nm; (+)-ESIMS *m*/*z* 231.1 [M + H]^+^; (+)-HRESIMS *m*/*z* 483.1274 [2M + Na]^+^ (calcd. For C_24_H_20_N_4_O_6_Na, 483.1275).

### 3.5. Antimicrobial Assays

Antimicrobial assays were performed according to the Antimicrobial Susceptibility Testing Standards outlined by the Clinical and Laboratory Standards Institute (CLSI) against *S. aureus* ATCC 6538, MRSA ATCC 29213, *E. coli* ATCC 11775, *P. aeruginosa* ATCC 15692, and *C. albicans* ATCC 10231 based on a 96-well microplate format in liquid growth. Briefly, glycerol stocks of the bacteria were inoculated on LB agar plate and cultured overnight at 37 °C. Glycerol stock of *C. albicans* was prepared on Sabouraud dextrose agar at 28 °C for 24 h. A single colony was picked and resuspended, then adjusted to approximately 10^4^ CFU/mL with Mueller-Hinton Broth for the bacteria and RPMI 1640 for the fungal suspension, respectively. Two μL of two-fold serial dilution of each compound (in DMSO) were added to each row on the 96-well microplate, containing 78 μL of microbe suspension in each well. Vancomycin and ciprofloxacin were used as positive controls for bacteria; Amphotericin B was used as positive for fungi; and DMSO was used as negative control. The final concentrations for the tested compounds were from 0.156 to 100 µg/mL by using two-fold diluted solutions. The 96-well plate of antibacterial was incubated at 37 °C aerobically for 16 h. The 96-well plate of antifungal was incubated at 35 °C aerobically for 24 h. Here, the MIC was defined as the minimum concentration of the compound that prevented visible growth of the tested bacteria.

### 3.6. Anti-Bacillus Calmette Guérin (BCG) Assay

The anti-BCG assays were carried out by using a constitutive GFP expression strain (pUV3583c-GFP) with direct readout of fluorescence as a measure of bacterial growth (isoniazid was used as positive control with MIC value of 0.05 µg/mL). The concentrations for the tested compounds were from 0.3125 to 40 µg/mL by using two-fold diluted solutions. The in vitro activity of compounds against BCG was determined in a 96-well plate as previously described [19].

## 4. Conclusions

Piperazine-triones belong to a class of rare alkaloids previously isolated from fungi [2,3,4,5,6,7,8,9,10] and derived from diketopiperazine characterized from *Streptomycetes* [20]. Currently eleven compounds containing piperazine-trione moiety have been reported. In our current study, two new piperazine-triones (**1**, **2**) were identified from a marine-derived *Streptomycetes* SMS636, together with four known secondary metabolites, lansai D (**3**), 1-*N*-methyl-(*E,Z*)-albonoursin (**4**), imidazo[4,5-*e*]-1,2,4-triazine (**5**) and streptonigrin (**6**). The two new piperazine-triones were proposed to be the produced when each of the double bonds for lansai D was oxidized. Compound **4** exhibited more potent antibacterial activities then those of **1**, **2** and **3**, which indicated the geometric configuration of the double bond between C-3 and C-5′ was important to the antibacterial bioactivities. Compound **6** exhibited significant antibacterial activities against *S. aureus*, MRSA and BCG with MIC values of 0.78, 0.78, and 1.25 μg/mL, which showed bioactivities similar to previously reported data [21]. The different structures of cell walls among gram positive bacteria, BCG, gram negative bacteria, and *C. albicans* may have resulted in the different sensitivity to **4** and **6.** These data highlight that secondary metabolites from marine actinomycetes are an excellent source of rare chemical entries in the fight against pathogenic bacteria.

## Figures and Tables

**Table 1:** NMR data for **1** and **2** (DMSO-*d*_6_).

Position	1		2	
	*δ*_H_, mult (*J* in Hz)	*δ*_C_	*δ*_H_, mult (*J* in Hz)	*δ*_C_
**2**		152.4		158.7
**3**		155.6		125.9
**4**	12.01, brs		11.44, s	
**5**		160.2		151.6
**6**		127.8		156.7
**7**	3.17, s	30.4	3.07, s	26.8
**1′**	5.81, d (9.0)	136.6	6.77, s	124.3
**2′**	3.94, m	26.3		133.7
**3′**	1.05, d (6.6)	22.7	7.53, d (7.8)	130.1
**4′**	1.05, d (6.6)	22.7	7.34, dd (7.8, 7.8)	127.7
**5′**			7.31, dd (7.8, 7.8)	128.3
**6′**			7.34, dd (7.8, 7.8)	127.7
**7′**			7.53, d (7.8)	130.1

**Table 2:** Antibacterial activity of **1**–**6** (μg/mL).

Compounds	*S. aureus* ^a^	MRSA ^a^	*E. coli* ^b^	*P. aeruginosa* ^b^	*BCG* ^c^	*C. albicans* ^d^
**1**	>100	>100	>100	>100	>40	>100
**2**	>100	>100	>100	>100	>40	>100
**3**	>100	>100	>100	>100	>40	>100
**4**	12.5	25	>100	>100	>40	>100
**5**	>100	>100	>100	>100	>40	>100
**6**	0.78	0.78	100	100	1.25	>100

^a^ Vancomycin was used as positive control with MIC value of 0.78 µg/mL; ^b^ Ciprofloxacin was used as positive control with MIC value of 0.78 µg/mL; ^c^ Isoniazid was used as positive control with MIC value of 0.05 µg/mL; ^d^ Amphotericin B was used as positive control with MIC value of 0.39 µg/mL.

## Supplementary Materials

The following are available online at https://www.mdpi.com/1660-3397/17/3/186/s1, Figures S1–S13: The UV, 1D and 2D NMR spectra of compounds **1** and **2**, and phylogenetic tree for *Streptomycetes* sp. SMS636.
