# Supplementary material for: Two New Piperazine-Triones from a Marine-Derived Streptomycetes sp. Strain SMS636
Source: Mar Drugs. 2019 Mar 21;17(3):186. doi: 10.3390/md17030186 (PMC6471054; doi:10.3390/md17030186)
Supplement: Supplementary file 1 [file marinedrugs-17-00186-s001.pdf]

marine drugs

Supplementary Materials

Two New Piperazine-Triones from a Marine-Derived *Streptomycetes* sp. Strain SMS636

Xiuli Xu [1], Jiahui Han [1,2], Rui Lin [1], Steven W. Polyak [3] and Fuhang Song [2,*]

[1] School of Ocean Sciences, China University of Geosciences, Beijing 100083, China; xuxl@cugb.edu.cn (X.X.); 15632779760@163.com (J.H.); linrui520@126.com (R.L.)

[2] CAS Key Laboratory of Pathogenic Microbiology and Immunology, Institute of Microbiology, Chinese Academy of Sciences, Beijing 100101, China

[3] School of Pharmacy and Medical Sciences, University of South Australia, Adelaide, 5000, Australia; steven.polyak@unisa.edu.au

* Correspondence: songfuhang@im.ac.cn; Tel.: +86-10-6480-6058

Content

Figure S1. ^{1}H NMR spectrum (600 MHz, DMSO-d_6) of **1** 3

Figure S2. ^{13}C NMR spectrum (150 MHz, DMSO-d_6) of **1** 3

Figure S3. HSQC spectrum (600 MHz, DMSO-d_6) of **1** 4

Figure S4. ^{1}H -^{1}H COSY spectrum (600 MHz, DMSO-d_6) of **1** 4

Figure S5. HMBC spectrum (600 MHz, DMSO-d_6) of **1** 5

Figure S6. ROESY spectrum (600 MHz, DMSO-d_6) of **1** 5

Figure S7. ^{1}H NMR spectrum (600 MHz, DMSO-d_6) of **2** 6

Figure S8. ^{13}C NMR spectrum (150 MHz, DMSO-d_6) of **2** 6

Figure S9. HSQC spectrum (600 MHz, DMSO-d_6) of **2** 7

Figure S10. ^{1}H -^{1}H COSY spectrum (600MHz, DMSO-d_6) of **2** 7

Figure S11. HMBC spectrum (600 MHz, DMSO-d_6) of **2** 8

Figure S12. ROESY spectrum (600 MHz, DMSO-d_6) of **2** 8

Figure S13. Neighbor-joining phylogenetic tree for *Streptomycetes* sp. strain SMS636 9

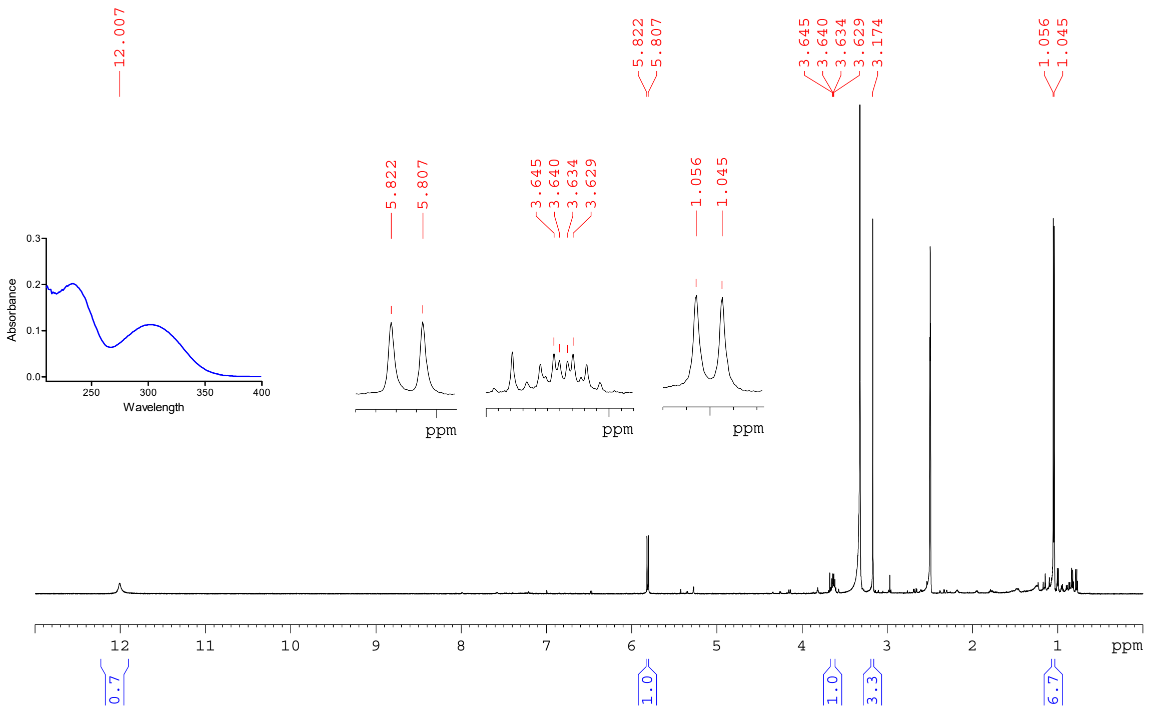

Figure S1. ^{1}H NMR spectrum (600 MHz, DMSO-d_6) of **1.**

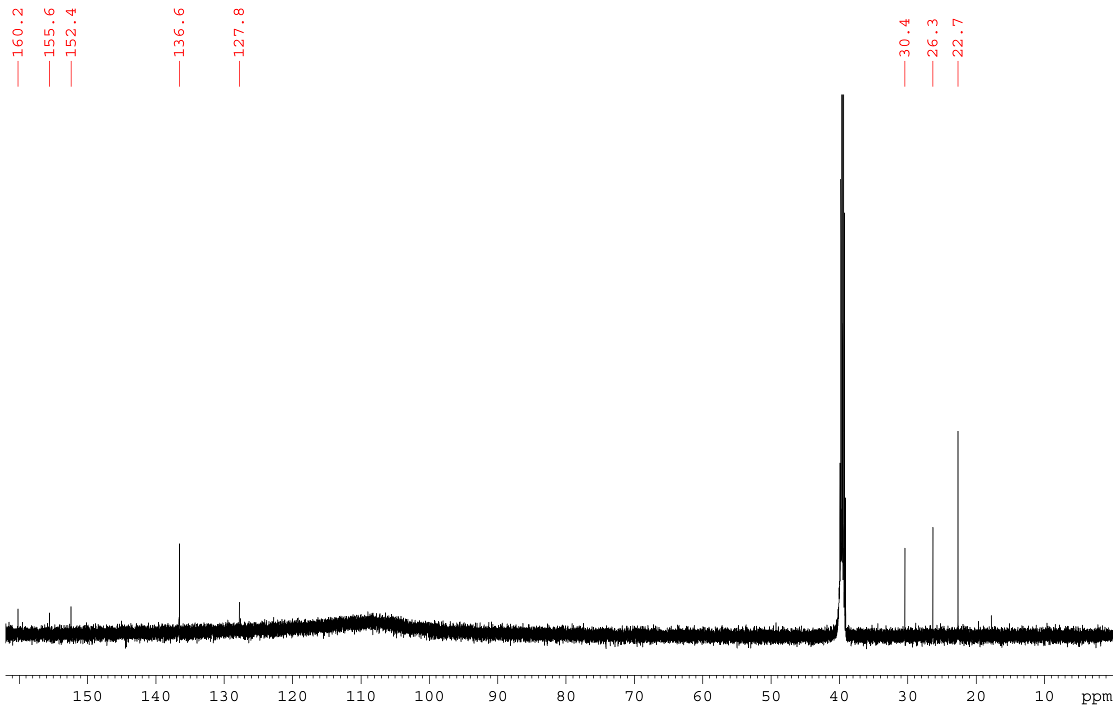

Figure S2. ^{13}C NMR spectrum (150 MHz, DMSO-d_6) of **1.**

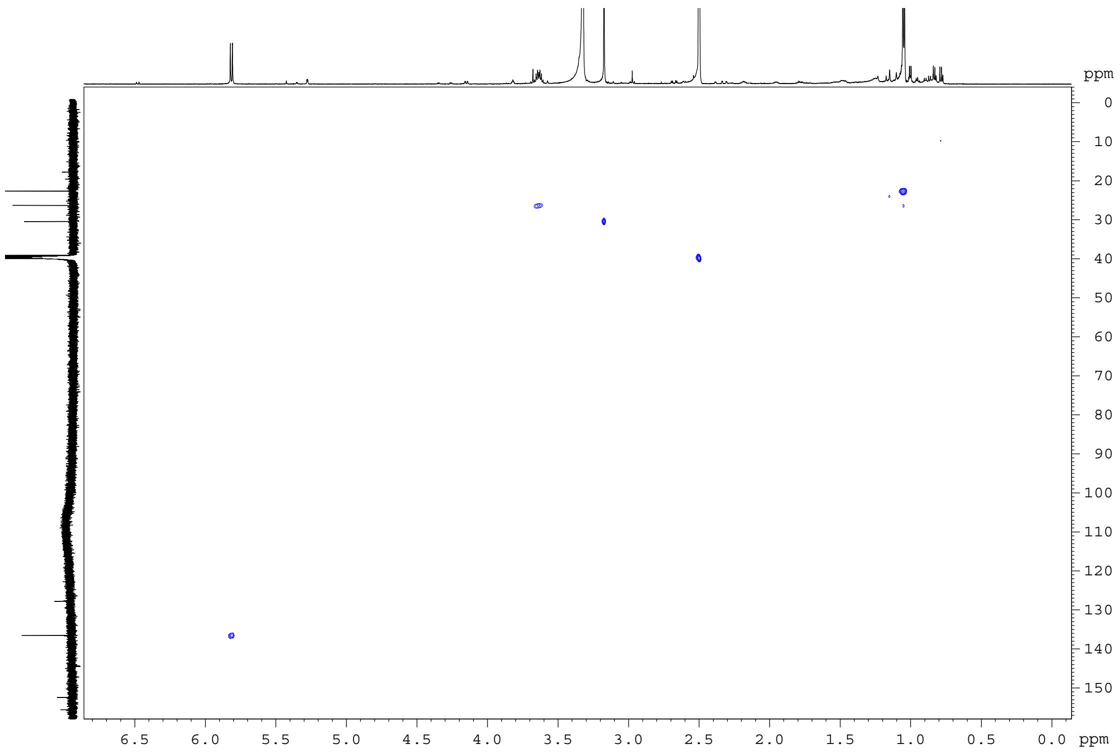

Figure S3. HSQC spectrum (600 MHz, DMSO-d_6) of **1.**

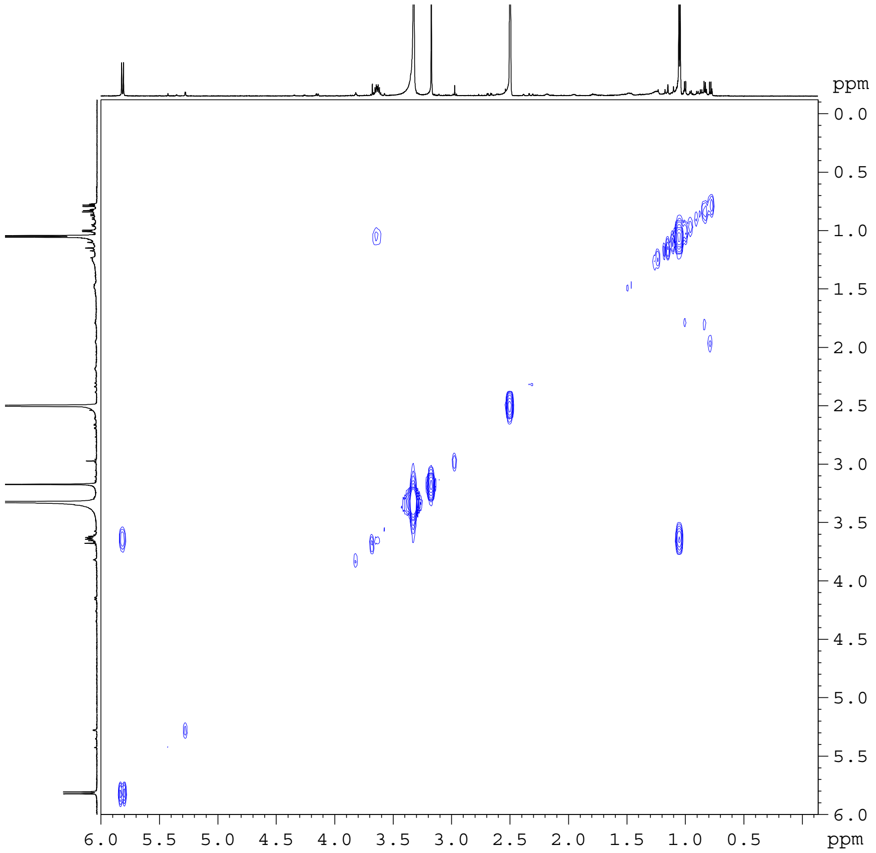

Figure S4. ^{1}H-^{1}H COSY spectrum (600 MHz, DMSO-d_6) of **1**.

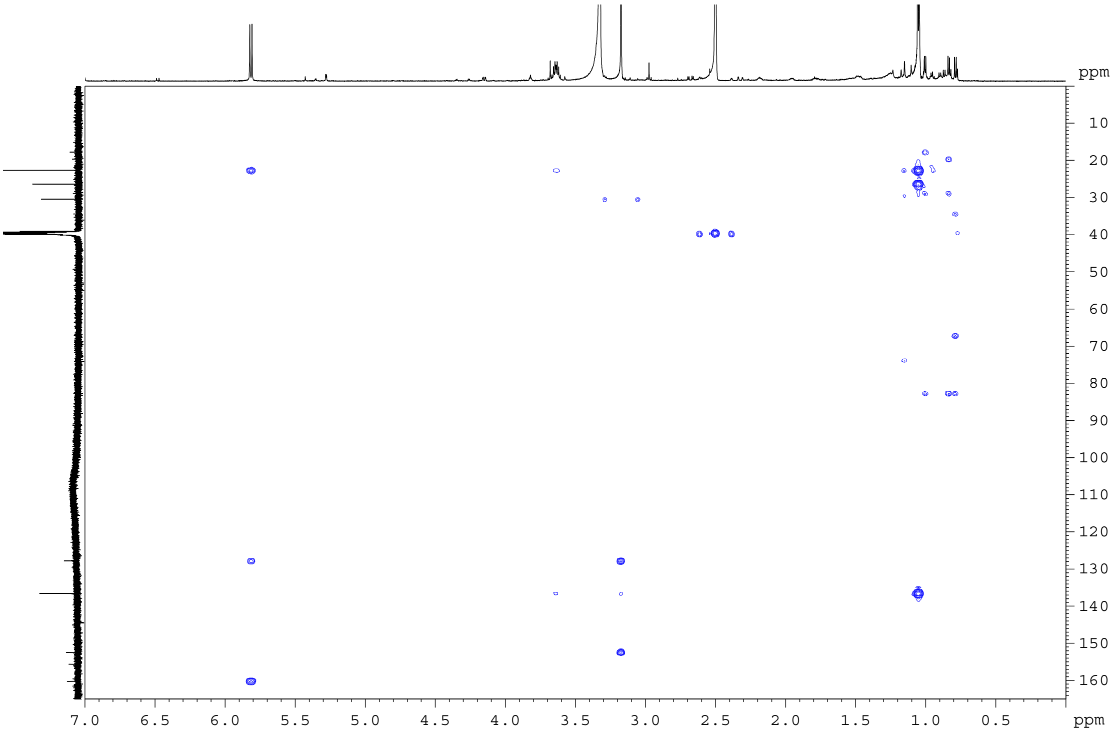

Figure S5. HMBC spectrum (600 MHz, DMSO-d_6) of **1**.

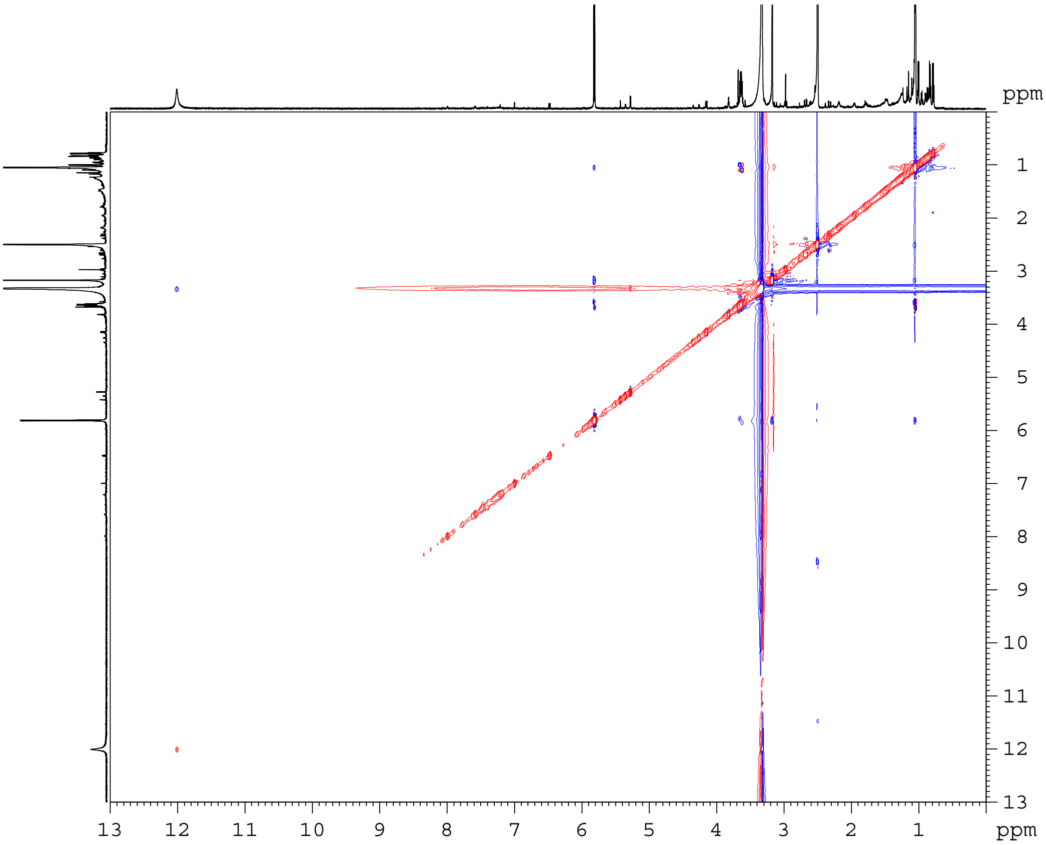

Figure S6. ROESY spectrum (600 MHz, DMSO-d_6) of **1**.

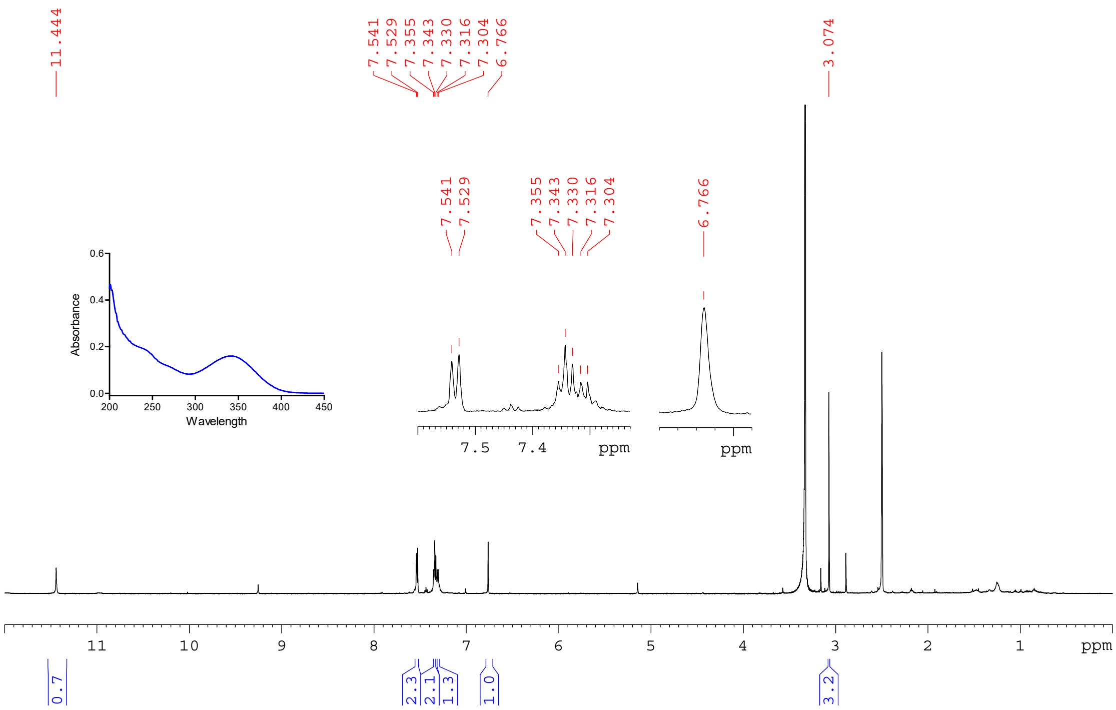

Figure S7. ^{1}H NMR spectrum (600 MHz, DMSO-d_6) of **2**.

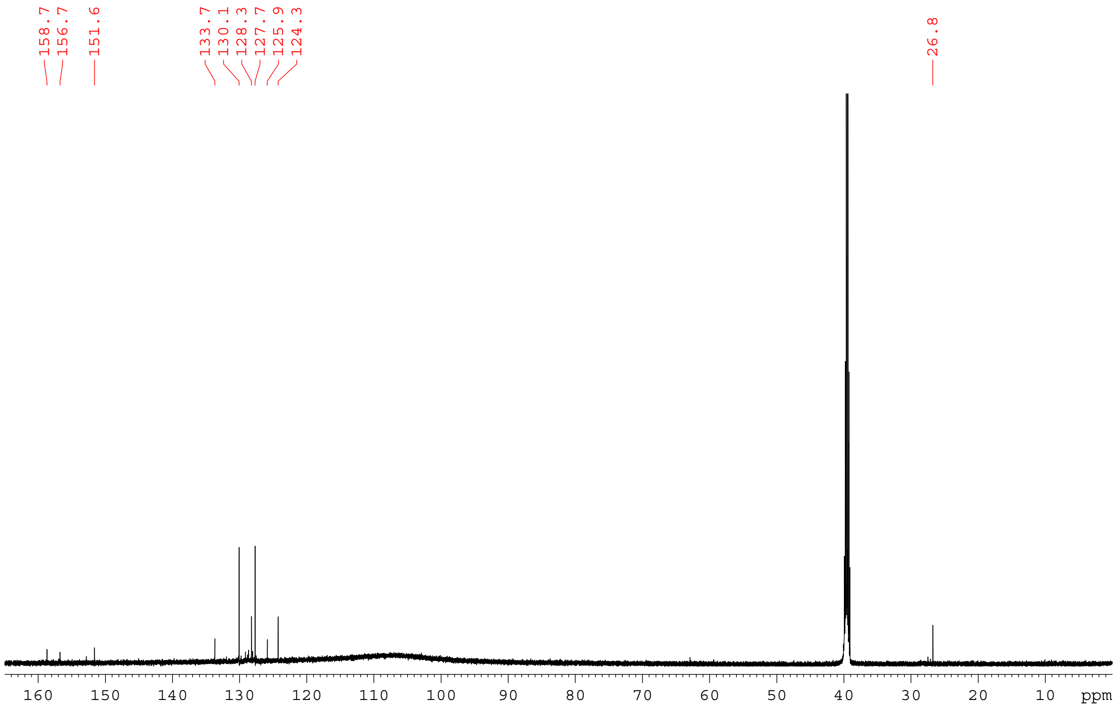

Figure S8. ^{13}C NMR spectrum (150 MHz, DMSO-d_6) of **2**.

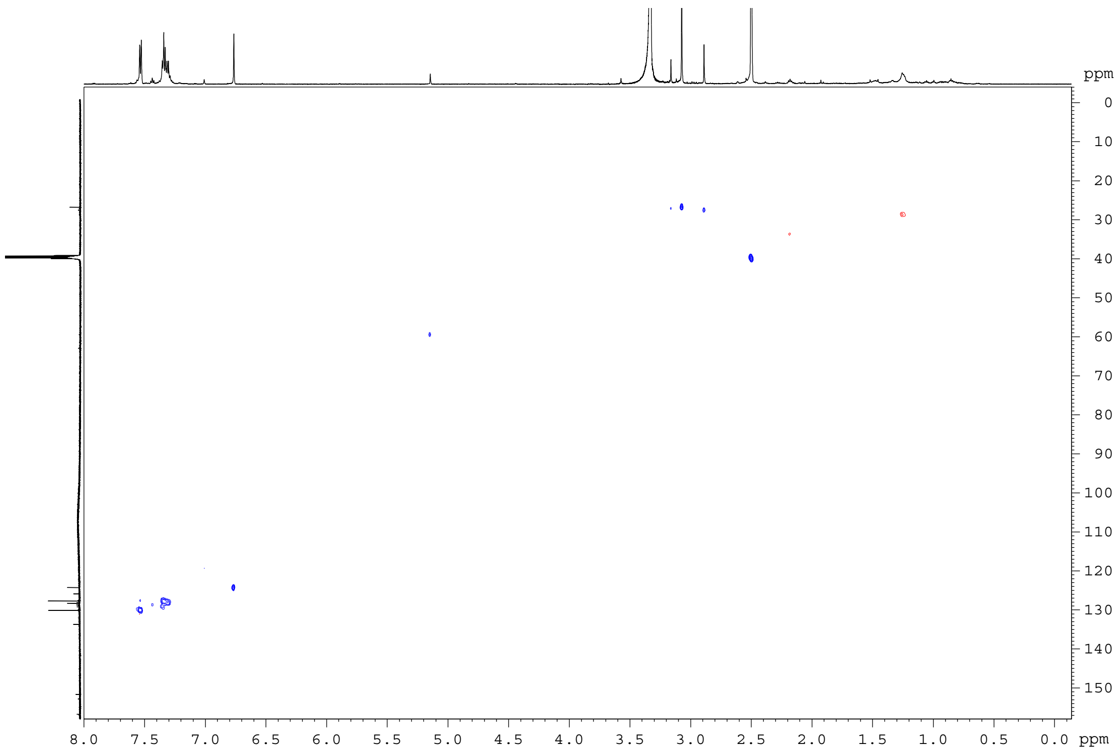

Figure S9. HSQC spectrum (600 MHz, DMSO-d_6) of **2**.

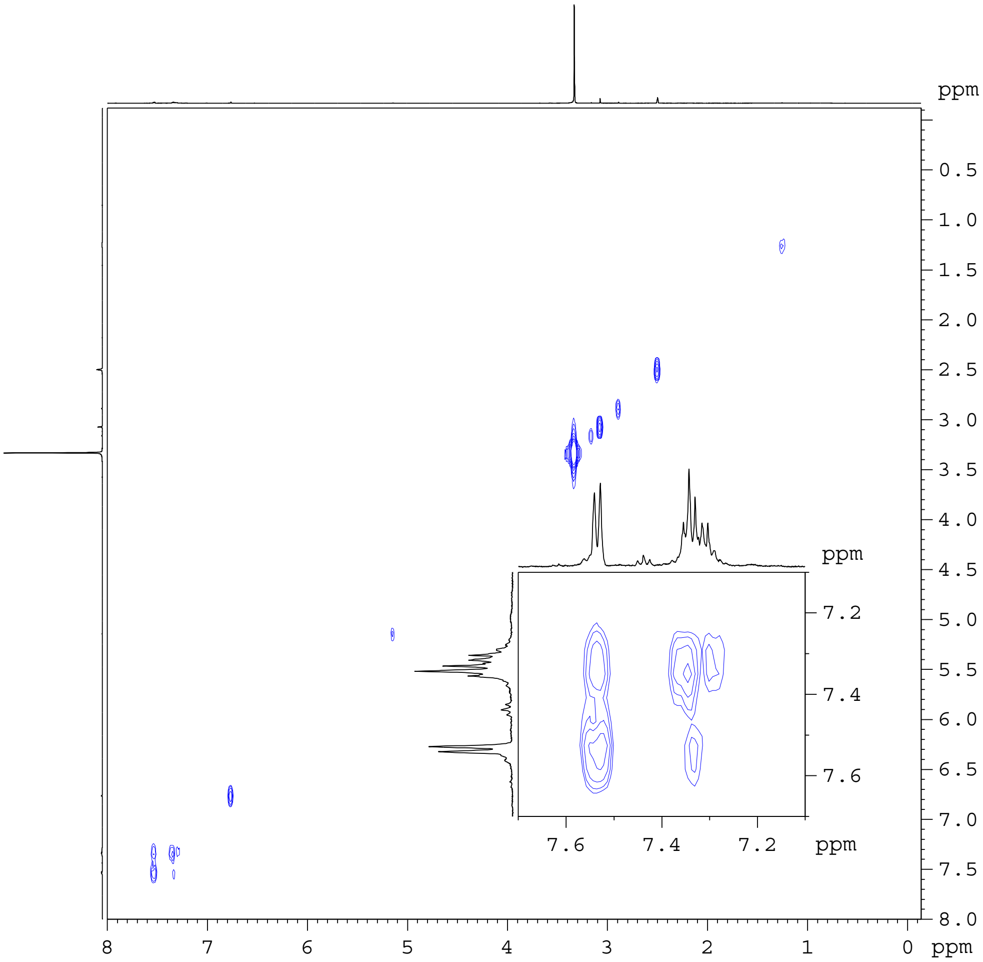

Figure S10. ^{1}H-^{1}H COSY spectrum (600MHz, DMSO-d_6) of **2**.

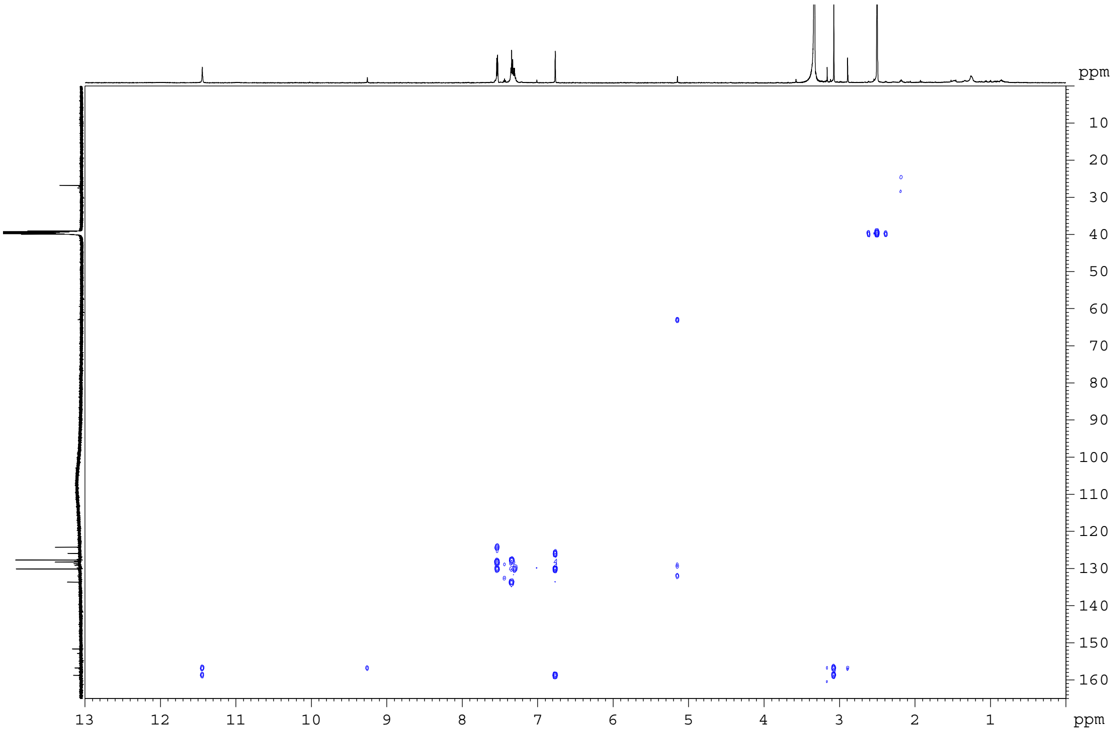

Figure S11. HMBC spectrum (600 MHz, DMSO-d_6) of **2**.

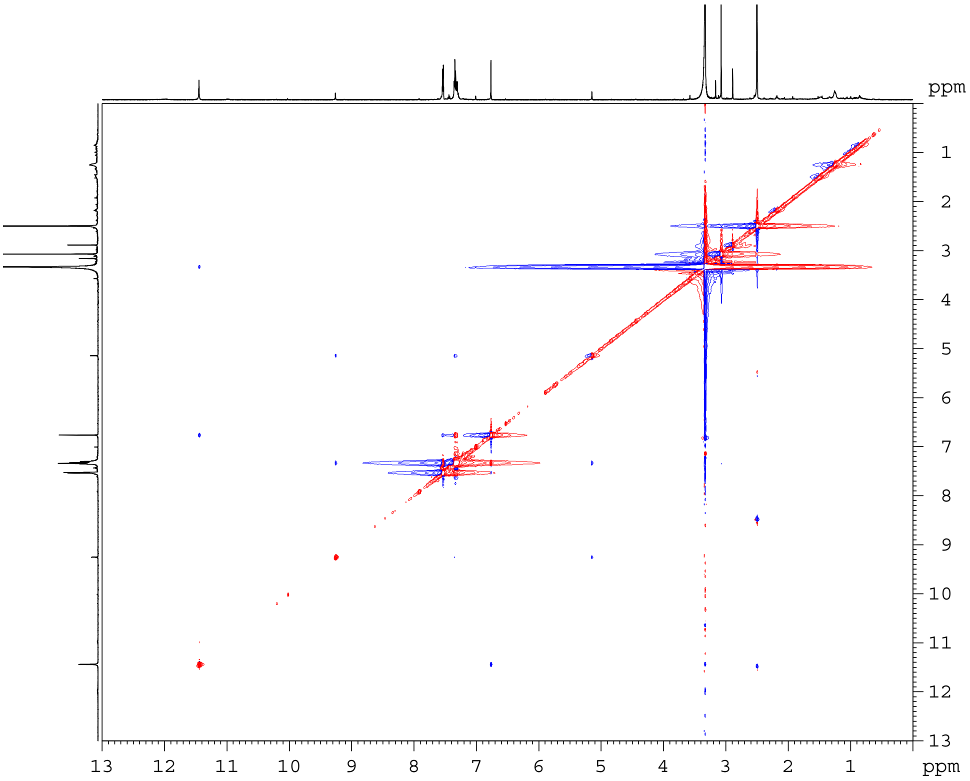

Figure S12. ROESY spectrum (600 MHz, DMSO-d_6) of **2**.

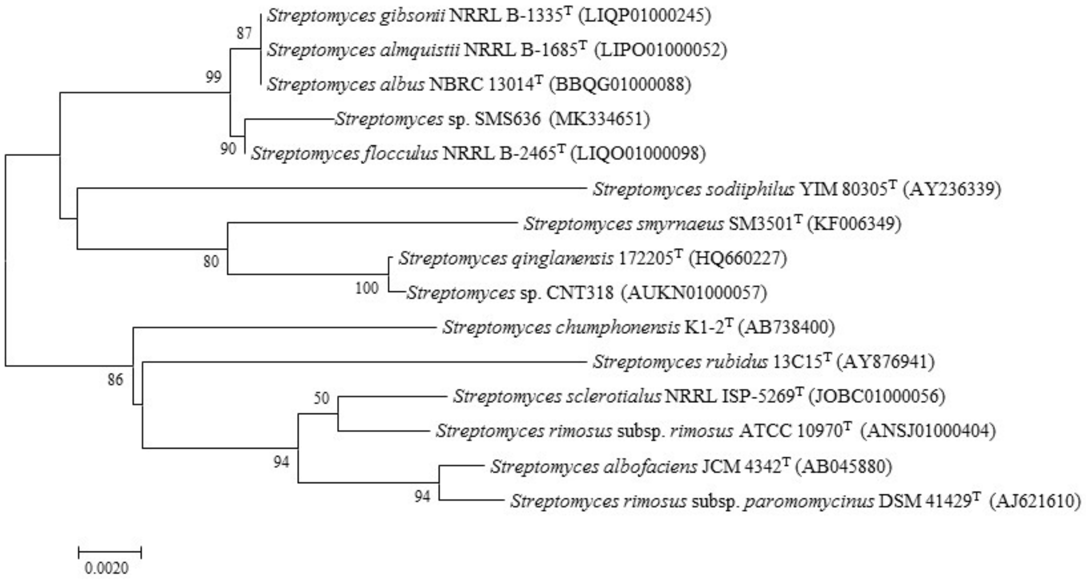

Figure S13. Neighbor-joining phylogenetic tree for *Streptomycetes* sp. strain SMS636. Only bootstrap values greater than 50% are shown (1000 resamplings) at nodes. DDBJ/EMBL/GenBank accession numbers of 16S rRNA gene sequences are shown in parentheses. Bar, 0.002 nt substitutions per.
